# Long-term follow-up of telehealth-enabled behavioral treatment for challenging behaviors in boys with fragile X syndrome

**DOI:** 10.1186/s11689-022-09463-9

**Published:** 2022-09-30

**Authors:** Scott S. Hall, Arlette Bujanda Rodriguez, Booil Jo, Joy S. Pollard

**Affiliations:** 1 Department of Psychiatry and Behavioral Sciences, Stanford University School of Medicine, 401 Quarry Road, Stanford, CA 94305-5795 USA; 2 Behavior Change Institute, Oakland, USA

## Abstract

**Background:**

A significant proportion of boys with fragile X syndrome (FXS), the most common known genetic cause of intellectual disability, exhibit challenging behaviors such as aggression and self-injury that can cause significant distress to families. Recent evidence suggests that coaching caregivers to implement functional communication training (FCT) with their child via telehealth can help to ameliorate these behaviors in FXS. In the present study, we followed families who had participated in our previous randomized controlled trial of FCT to evaluate the longer-term effects of FCT on challenging behaviors in this population.

**Methods:**

In study 1, follow-up emails, phone calls, text messages, and letters were sent to caregivers of 48 boys with FXS who had completed our previous study conducted between 2016 and 2019. The main outcome measures administered at follow-up were the Aberrant Behavior Checklist–Community (ABC-C) and the Parenting Stress Index, 4th Edition (PSI-4). In study 2, families who had received FCT treatment but whose child exhibited challenging behaviors daily at follow-up received a 1-h parent training booster session to determine whether the intervention effect could be recovered.

**Results:**

Sixteen (66.7%) of 24 families who had received FCT treatment and 18 (75.0%) of 24 families who had received treatment as usual were traced and consented between March and August 2021. The mean follow-up time was 3.1 years (range, 1.4 to 4.2 years). Longitudinal mixed effects analyses indicated that boys who had received FCT were more likely to show improvements on the irritability and lethargy/social withdrawal subscales of the ABC-C over the follow-up interval compared to boys who had continued with treatment as usual. Four of the six boys who had received the booster parent training session via telehealth were reported to exhibit fewer forms of challenging behavior at a 4-week follow-up.

**Conclusions:**

Empowering parents to implement behavior analytic treatments with their child in their own home can have durable effects on maintaining low levels of challenging behaviors in boys with FXS. These data further support the need to implement parent-mediated interventions for challenging behaviors in this population at an early age.

**Trial registration:**

ClinicalTrials.gov, ^NCT03510156^. Registered 27 April 2018

## Introduction

A significant proportion of boys with fragile X syndrome (FXS), the most common known genetic cause of intellectual disability, exhibit challenging behaviors such as aggression, self-injury, and property destruction that can cause significant distress to caregivers and interfere with the child’s educational and social development [1–3]. Prevalence studies suggest that challenging behaviors occur in approximately 60–80% of boys with FXS, a significantly higher prevalence than expected for children with similar levels of intellectual disability in general [4–6]. These behaviors can result in bruising, bleeding, and other tissue damage and can often require medical attention. Caring for a child with FXS who exhibits challenging behavior places an increased burden on the family, resulting in poor child outcomes and decreased quality of life for the child and family as well as high medical and social costs [7, 8]. Given that challenging behaviors are likely to begin in childhood and persist into adulthood, interventions to ameliorate these behaviors early in the child’s life would appear to be particularly important.

FXS affects approximately 1 in 3000 to 4000 boys and 1 in 6000 to 8000 girls and is caused by mutations to the fragile X messenger ribonucleoprotein 1 (*FMR1*) gene on the X chromosome at region q27.3 [9]. Hypermethylation of the gene results in reduced or absent expression of fragile X messenger ribonucleoprotein (FMRP), a key protein involved in synaptic pruning and dendritic maturation in the brain [10]. Over the past few decades, treatments for challenging behaviors in FXS have typically involved pharmacological agents (e.g., antipsychotics, stimulants), as well as experimental pharmacotherapies targeted to the downstream effects of reduced FMRP [11]. However, these approaches have not been successful in ameliorating challenging behaviors in children with FXS [11–16]. Furthermore, there are no FDA-approved pharmacological treatments for FXS. Notably, in recent surveys of treatment priorities identified by caregivers of children with FXS and other key stakeholders, challenging behaviors were rated as one of the most significant areas of concern for families [17, 18]. However, with the exception of our recently completed randomized controlled trial [3], evidence-based behavioral interventions to ameliorate or prevent challenging behaviors in FXS have not been forthcoming.

In our previous randomized controlled trial, we examined whether a behavior analytic intervention called functional communication training (FCT) administered via telehealth could ameliorate challenging behaviors in boys with FXS, ages 3 to 10 years [3]. FCT involves first identifying the potential social reinforcers that may be maintaining the child’s challenging behavior and then teaching the child to emit an alternative functional communication response to gain the same source of reinforcement more appropriately. For example, if a child typically engages in challenging behavior to escape from demanding tasks, the focus of the treatment would be to teach the child to emit an alternative communication response (e.g., “break”) in order to remove task demands and simultaneously ensuring that task demands are not removed if the child engages in challenging behavior. To expand the reach of the intervention, the FCT intervention was implemented via telehealth. Specifically, parents of boys assigned to the FCT treatment group were coached remotely over Zoom by a Board Certified Behavior Analyst (BCBA) to implement the procedures directly with their child in their own home. Parents of boys randomized to the treatment as usual group continued their child's treatment as usual including any medications or additional therapies, such as speech therapy or occupational therapy. The results of the study showed that boys with FXS who received FCT via telehealth showed significantly greater decreases on the irritability subscale of the Aberrant Behavior Checklist–Community (ABC-C) [19], our primary outcome measure, compared to children who had continued with treatment as usual. In addition, levels of parenting stress were also significantly decreased on subscales of the Parenting Stress Index, 4th Edition [20] related to the child’s behavior for parents of boys who had received FCT via telehealth compared to boys who had continued with treatment as usual.

Although the FCT intervention showed promising results over the 12-week treatment period, it would be important to better understand the longer-term benefits of delivering behavioral treatments via telehealth for this population. In study 1, we re-contacted families who had completed our previous study and examined levels of challenging behavior and family stress at follow-up. For participants who had received FCT treatment but still exhibited daily challenging behavior at follow-up, in study 2, we examined whether delivering an additional booster therapy session via telehealth might help to reestablish the treatment’s effectiveness. A single booster session was delivered via telehealth to reacquaint families with the treatment procedures and outcomes were measured before and after the booster at a 4-week follow-up.

### Study 1

#### Methods

In our previous RCT study conducted between 2016 and 2019, boys with FXS were included if they had a confirmed genetic diagnosis of FXS (> 200 CGG repeats on the *FMR1* gene with evidence of aberrant methylation), were aged between 3 and 10 years inclusive, and exhibited at least one form of challenging behavior daily on the Behavior Problems Inventory–Short Form (BPI-S) [21]. Because the intervention was conducted via telehealth, families were required to have internet service at home with a signal that could host video-streaming capability. Families were excluded from the study if the child had a significant sensory impairment (e.g., blindness or deafness), a neurological condition (e.g., frequent seizures, brain injury, Tourette’s syndrome) or if they received applied behavior analysis (ABA) services in excess of 5 h per week. Throughout the study, caregivers were asked to ensure that their child’s other therapies (i.e., medications or other treatments) remained as stable as possible.

For the present study, caregiver-child dyads who had completed the previous study in each group (24 families in the FCT group and 24 families in the treatment as usual group) were contacted in the order in which they were previously enrolled via phone, email invitations, or text messages. If this was unsuccessful, a letter was sent to the address on file inviting them to participate in the study. All invitations were made from March 2021 to August 2021.

##### Outcome measures

The *Aberrant Behavior Checklist-Community* (ABC-C) [19] is a 58-item parent-report measure of problem behaviors commonly exhibited by children and adults with developmental disabilities. Items on the ABC-C are rated on a 4-point severity scale from 0 to 3 and resolve into five subscales: irritability (15 items), lethargy/social withdrawal (16 items), stereotypic behavior (7 items), hyperactivity/non-compliance (16 items), and inappropriate speech (4 items). The alpha coefficients for the five subscales of the ABC-C range from .86 to .94. In the previous RCT study, the irritability subscale of the ABC-C was employed as the primary outcome measure because it contains items relating to aggression, self-injury, tantrums, agitation, and unstable mood and is often employed in clinical trials. The standard scoring algorithm was used rather than the fragile X scoring algorithm [21] so that the results would be comparable to other published studies involving children with autism spectrum disorder (ASD).

The *Parenting Stress Index, 4th Edition* (PSI-4) [20] is a 120-item instrument designed to measure parent stress related to the parent and the child’s behavior. Items are divided into two main domains: *child domain* and *parent domain*, which combine to form a *total stress* scale. Scores are reported as T-scores with a mean of 50 and a standard deviation of 10. Reliability coefficients for this measure range from .78 to .88. This measure is the most widely used measure of parental stress in the literature and has been employed as an outcome measure in a large number of clinical trials, including our own recently published RCT.

Finally, the Behavior Problems Inventory–Short Form (BPI-S) [22] was administered to determine whether children exhibited challenging behavior on a daily basis. The scale contains 30 items: self-injurious behavior (8 items), aggressive/destructive behavior (10 items), and stereotyped behavior (12 items) with each item rated on a five-point frequency scale (never = 0; monthly = 1; weekly = 2; daily = 3; hourly = 4). Boys with FXS exhibited daily challenging behavior if they received a score of 3 or 4 on at least one of the self-injurious behavior or aggressive/destructive behavior items. All behavioral rating forms were completed by the same primary caregiver who had participated in our previous RCT. Table 1 shows the demographic and clinical characteristics of the families at baseline in each group. There were no differences in age, adaptive behavior, or scores obtained on the ABC-C and PSI-4 at baseline between the two groups. Just over two-thirds of boys in each group were taking psychotropic medications.

**Table 1:** Demographic and clinical characteristics of participants in study 1 at baseline

Measure	FCT via telehealth (*n* = 24)	Treatment as usual (*n* = 24)
Child age in years (M, SD)	6.68 (2.22)	7.21 (2.19)
Adaptive behavior age equivalent in years (M, SD)^a^		
Receptive communication	2.41 (1.42)	2.82 (1.46)
Expressive communication	2.42 (1.23)	2.77 (1.04)
ABC-C^b^ (M, SD)		
Irritability	19.46 (8.66)	17.13 (9.62)
Lethargy/social withdrawal	8.25 (7.48)	5.67 (7.20)
Stereotypic behavior	6.58 (4.80)	5.08 (3.68)
Hyperactivity/non-compliance	22.54 (10.89)	21.58 (9.06)
Inappropriate speech	3.50 (3.32)	3.79 (2.93)
PSI-4^c^ (M, SD)		
Child domain	64.88 (6.62)	63.79 (7.49)
Parent domain	53.46 (8.07)	55.29 (9.52)
Total stress	59.08 (6.79)	59.71 (8.37)
Other therapies (%)		
Psychotropic medications	68.8%	66.7%
Speech therapy	75.0%	62.5%
Occupational therapy	79.2%	45.8%
Physical therapy	50.0%	20.8%
Behavior therapy (including ABA)	8.3%	4.2%

^a^Vineland Adaptive Behavior Scales, 3rd Edition

^b^Aberrant Behavior Checklist–Community

^c^Parenting Stress Index, 4th Edition

##### Statistical analysis

For the analyses of longitudinal data, we used standard mixed effects modeling [23, 24], treating individual age at the two assessment points as time. We assumed a linear trend for all key outcomes and allowed individual variations in terms of age of assessment, their outcomes at baseline (random intercept), and how their outcomes changed (random slope) as they aged. For maximum likelihood estimation of our mixed effects models, we used Mplus version 8.6 [25], an advanced latent modeling program. In line with the intention to treat principle, we utilized all available cases as long as they had at least one outcome measure. The attrition (missing data) rate at the follow-up assessment was 33.3% in the FCT treatment group and 25.0% in the treatment as usual group. We assumed that data were missing at random conditional on observed data [26], a well-accepted practical approach in modern longitudinal analysis. Mixed effects modeling results were summarized in terms of yearly changes and group differences in yearly changes. We consistently used an unadjusted significance level (α = .05, two-tailed) in all analyses without correcting for multiple comparisons given the preliminary nature of our study. For within group changes, Cohen’s *d* effect sizes were calculated by dividing the estimated slope by the baseline sample standard deviation. For group differences, effect sizes were calculated by dividing the estimated slope difference by the baseline sample standard deviation pooled across the two groups. Finally, to examine whether children who were assessed after a shorter time lapse exhibited fewer challenging behaviors, we computed correlations between change per year on the subscales of the ABC-C and time between baseline and follow-up.

### Results

Of the 48 families who completed the previous study, we were able to contact 43 families by phone, email, or text. Two families subsequently decided not to participate in the study and so 41 families were sent consent forms. Five families did not return the consent form and two signed consent but subsequently withdrew from the study. Therefore, we obtained follow-up data for 34 families, 16 of whom had previously received FCT treatment and 18 of whom had received treatment as usual. The mean follow-up time was 2.8 years (range = 1.4 to 4.2 years) for participants who had received FCT treatment and 3.2 years (range = 2 to 4.2 years) for participants who had received treatment as usual.

**Fig. 1:**
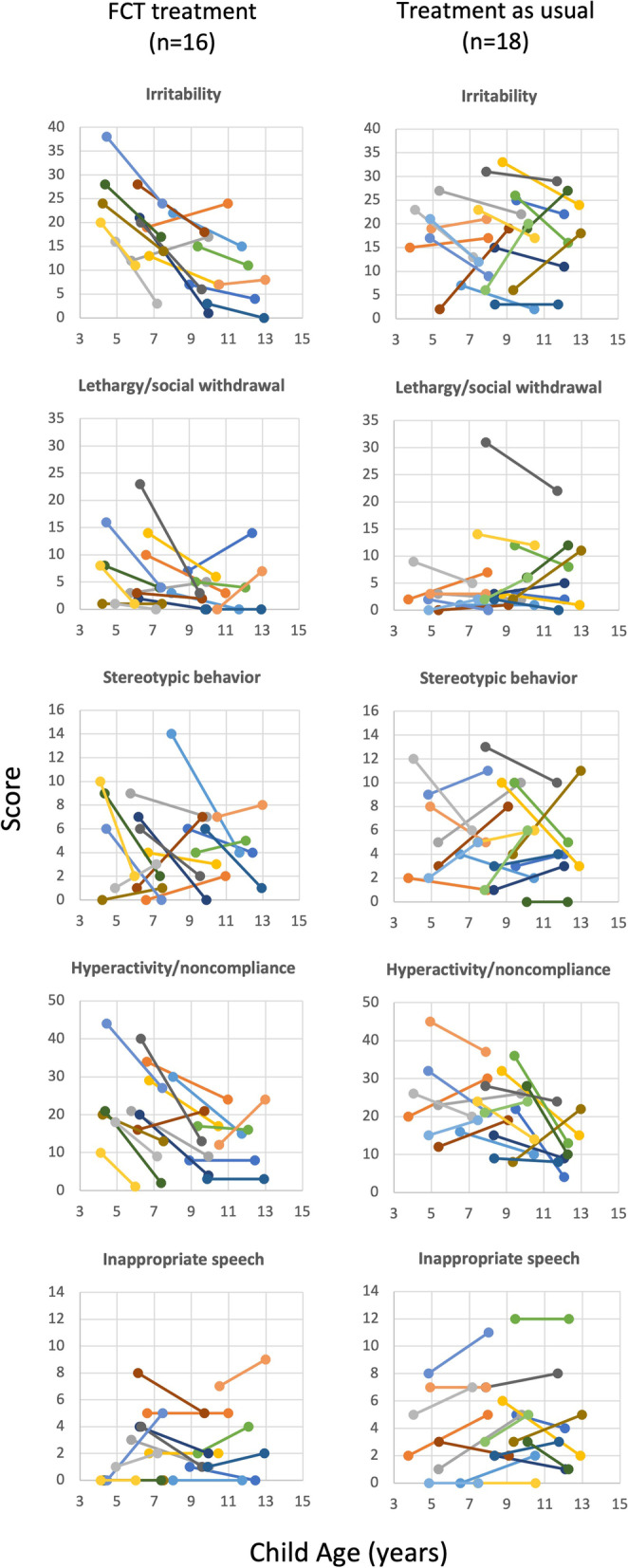
Trajectory of scores obtained at baseline and follow-up on each subscale of the Aberrant Behavior Checklist–Community (ABC-C) plotted by age for boys in each group

Figure 1 shows the scores obtained on each subscale of the ABC-C from baseline to follow-up for boys in each group. In each graph, the trajectory of scores for each individual participant is plotted over time by child age. Table 2 shows within-group and between-group differences in scores from baseline to follow-up based on mixed effects modeling.

**Table 2:** Within and between-group differences based on mixed effects modeling for each outcome in study 1

Outcome		FCT via telehealth (*n* = 24)	Treatment as usual (*n* = 24)	Group difference
ABC-C^a^				
Irritability	Change (per year)	− 1.916	0.068	− 1.984
	95% CI of change	(− 2.778, − 1.055)	(− 0.685, 0.821)	(− 3.110, − 0.859)
	Effect size (per year)	− 0.22	0.01	− 0.23
	*p* value (2-tailed)	0.000	0.859	0.001
Lethargy/social withdrawal	Change (per year)	− 1.006	0.129	− 1.135
	95% CI of change	(− 1.798, − 0.214)	(− 0.374, 0.632)	(− 2.107, − 0.163)
	Effect size (per year)	− 0.13	0.02	− 0.15
	*p* value (2-tailed)	0.013	0.615	0.022
Stereotypic behavior	Change (per year)	− 0.487	0.026	− 0.513
	95% CI of change	(− 0.970, − 0.004)	(− 0.343, 0.396)	(− 1.126, 0.099)
	Effect size (per year)	− 0.10	0.01	− 0.12
	*p* value (2-tailed)	0.048	0.890	0.100
Hyperactivity/non-compliance	Change (per year)	− 2.001	− 1.046	− 0.955
	95% CI of change	(− 3.085, − 0.918)	(− 1.997, − 0.094)	(− 2.371, 0.460)
	Effect size (per year)	− 0.18	− 0.12	− 0.10
	*p* value (2-tailed)	0.000	0.031	0.186
Inappropriate speech	Change (per year)	− 0.057	0.192	− 0.249
	95% CI of change	(− 0.358, 0.245)	(− 0.045, 0.429)	(− 0.627, 0.130)
	Effect size (per year)	− 0.02	0.07	− 0.08
	*p* value (2-tailed)	0.712	0.112	0.197
PSI-4^b^				
Child domain	Change (per year)	− 1.276	0.059	− 1.334
	95% CI of change	(− 2.150, − 0.401)	(− 0.818, 0.936)	(− 2.557, − 0.112)
	Effect size (per year)	− 0.19	0.01	− 0.19
	*p* value (2-tailed)	0.004	0.895	0.032
Parent domain	Change (per year)	− 0.909	0.691	− 1.600
	95% CI of change	(− 2.081, 0.263)	(− 0.060, 1.443)	(− 2.985, − 0.215)
	Effect size (per year)	− 0.11	0.07	− 0.18
	p-value (2-tailed)	0.128	0.072	0.024
Total stress	Change (per year)	− 1.060	0.510	− 1.569
	95% CI of change	(− 1.992, − 0.127)	(− 0.244, 1.263)	(− 2.736, − 0.403)
	Effect size (per year)	− 0.16	0.06	− 0.21
	*p* value (2-tailed)	0.026	0.185	0.008

^a^Aberrant Behavior Checklist–Community

^b^Parenting Stress Index, 4th Edition; CI = confidence interval

On the irritability subscale of the ABC-C, the mean change per year was − 1.92 points (CI_95%_ = − 2.778, − 1.055) for boys who had received FCT and was .068 points (CI_95%_ = − 0.685, 0.821) for boys who had received treatment as usual, a significant difference between the groups (*p* = .001, *d* = − .23). On the lethargy/social withdrawal subscale, the mean change per year was − 1.006 points (CI_95%_ = − 1.798, − 0.214) for boys who had received FCT and was .129 points (CI_95%_ = − 0.374, 0.632) for boys who had received treatment as usual, a significant difference between the groups (*p* = .022, *d* = − .15). These data indicated that scores decreased at a faster rate on the irritability and lethargy/social withdrawal subscales from baseline to follow-up for those who had received FCT treatment compared to those who had received treatment as usual. There were no other significant differences between the groups in the trajectory of scores on the other subscales of the ABC-C. There was a significant correlation between change per year on the irritability subscale of the ABC-C and time between baseline and follow-up (*r*(16) = − .495, *p* = .026, one tailed) indicating that the treatment gains were stronger closer to the initial treatment.

**Fig. 2:**
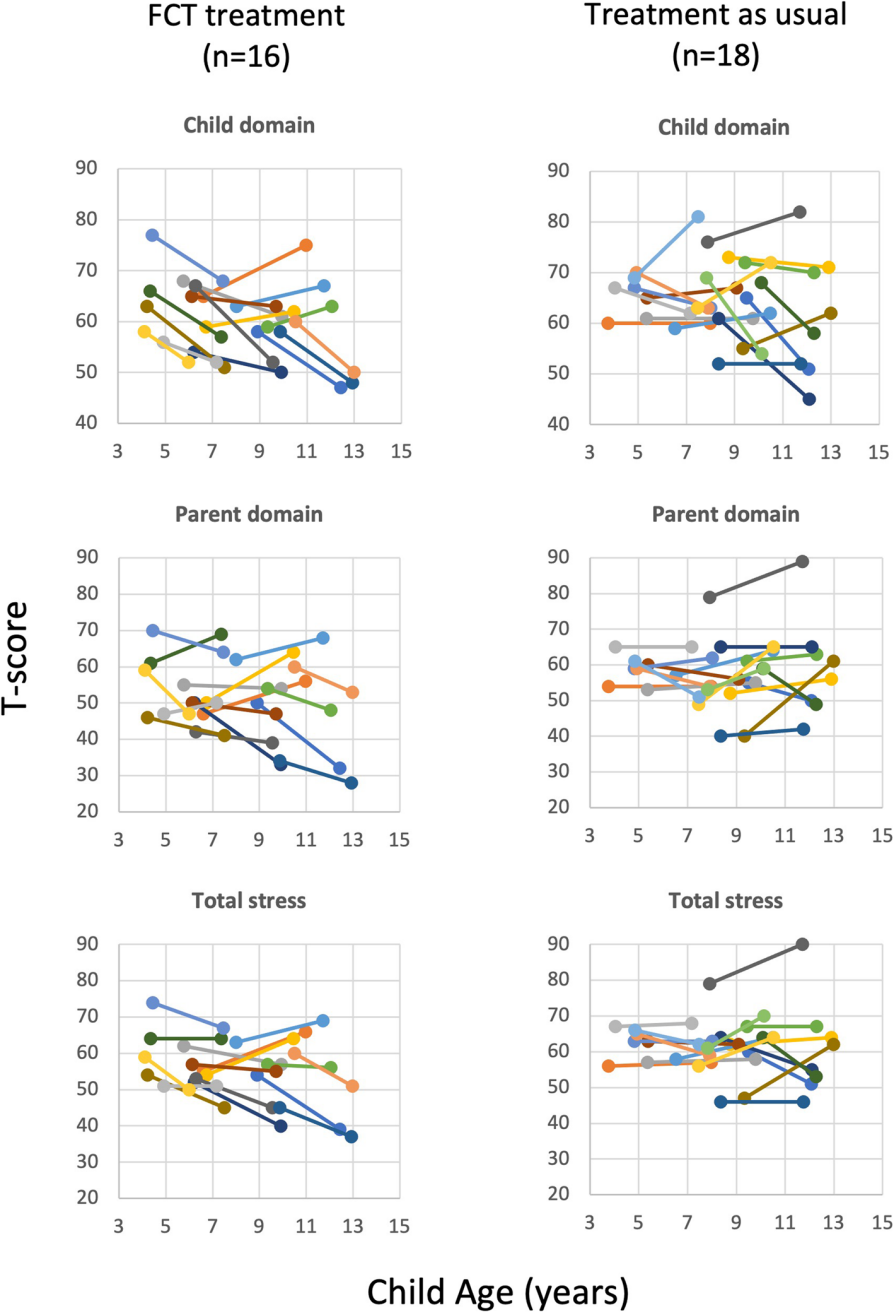
Trajectory of scores obtained at baseline and follow-up on each subscale of the Parenting Stress Index-4th Edition (PSI-4) plotted by age for boys in each group

Figure 2 shows the scores obtained on each subscale of the PSI-4 from baseline to follow-up for boys in each group. As before, in each graph, the trajectories of scores for each individual participant are plotted over time by child age. The corresponding within-group and between-group differences in scores from baseline to follow-up based on mixed effects modeling are shown in Table 2.

On the child domain, the mean change per year was − 1.276 points (CI_95%_ = − 2.150, − 0.401) for boys who had received behavioral treatment and was .059 points (CI_95%_ = − 0.818, 0.936) for boys who had received treatment as usual, a significant difference between the groups (*p* = .032, *d* = − .19). There were also significant differences between the groups on the parent domain and the total stress scores of the PSI-4. These data indicated that parenting stress decreased at a faster rate from baseline to follow-up for those who had received FCT treatment compared to those who had received treatment as usual. There were no differences from baseline to follow-up in terms of the proportion of children who were receiving psychotropic medications, speech therapy, occupational therapy, or physical therapy in either group. Five of 16 (31.3%) boys who had received FCT and 4 of 18 (22.2%) boys who had received treatment as usual were reported to be receiving ABA therapy at follow-up.

On the BPI-S, 11 of 16 (68.8%) boys who had received FCT and 14 of 18 boys (77.8%) who received treatment as usual were reported to display at least one form of challenging behavior daily at follow-up. Twelve (75.0%) boys who had received FCT and 15 (83.3%) boys who had received treatment as usual were also taking psychotropic medications at follow-up. A chi-square analysis indicated that boys who displayed daily challenging behaviors were significantly more likely to be taking psychotropic medications (*χ*^2^(1) = 9.15, *p* = .002)

### Study 2

#### Methods

##### Participants

The 11 parent-child dyads with FXS who had received FCT treatment and exhibited at least one form of daily challenging behavior at follow-up on the BPI-S were eligible to participate in study 2. Four of the eleven families declined to participate in study 2 because they said the booster session was unnecessary, and one family withdrew. Six families, therefore, participated in study 2 and received the booster session.

P10 was an 11-year-old male who displayed four topographies of challenging behavior daily (hitting others, kicking others, grabbing others, and destroying things). As a result of his property destruction, P10 had made several holes in the walls throughout his house. P10 used vocal language to communicate, with full sentences of 3−5 words and was enrolled in a special education school where he was able to attend until the COVID-19 pandemic began. During the pandemic, P10 was homeschooled with an in-home babysitter who also became his primary caregiver. P10 had begun to display challenging behavior primarily due to constantly being at home and not being able to go to the park, walks or shopping for toys. At this time, his family would often order items online and this led P10 to experience higher levels of anxiety waiting for delivery vehicles which often resulted in challenging behavior. P10’s parents created a padded area in the house for him to de-escalate during severe aggressive episodes. Sometimes P10 would transition himself independently to calm down in this area, but sometimes he would require physical guidance from his parents.

P14 was a 9-year-old male who displayed two forms of challenging behavior daily (self-biting and head-hitting). He used vocal language to communicate with full sentences of 5–6 words and attended school in a special classroom in person prior to the pandemic. During the pandemic, P14 began attending school and speech therapy online and began displaying problem behavior by refusing to attend school if his mother was not present to consistently redirect him to attend to the computer screen. P14 also began displaying inappropriate noises during online schooling. In addition, he began biting his arm and hitting himself to get attention from his peers online. He was reported to experience higher anxiety levels when he and his family needed to go out of the house into the community. For example, whenever P14 had to attend doctor’s appointments in-person or go grocery shopping with his mother without knowing what to expect, this would often lead to outbursts of self-stimulatory behavior (swinging his arms above his head). His parents began redirecting this behavior to put his arms down, leading to self-injurious behavior.

P23 was an 11-year-old male who displayed four forms of challenging behavior (self-biting, head-hitting, hitting others and grabbing others). He used an augmentative and alternative communication (AAC) device (Proloquo2Go) on an iPad to communicate, being able to build sentences of three words on the software (e.g., selecting three different icons on the AAC device to request “I want more time please”). Prior to the pandemic, P23 attended school in a special education classroom for children with complex needs. P23 began online school when the COVID-19 pandemic started and this transition had been difficult for him and he had begun displaying challenging behavior daily.

P25 was a 13-year-old male who displayed six forms of challenging behavior daily. He used vocal language with full sentences of 3–5 words and was attending school in a special education classroom prior to the pandemic. During the pandemic, P25 stopped receiving speech therapy and began attending school at a reduced level for only a couple of hours per day online. P25 often experienced anxiety levels due to being home with low structure and low activity throughout the day, and he often displayed challenging behavior by biting his knuckles. As P25’s day structure began including more activities throughout the day, he began displaying daily levels of self-biting, particularly when he had to transition across activities.

P32 was a 9-year-old male who displayed two forms of challenging behavior on a daily. He used vocal language with full sentences of 6+ words and attended school in a general education classroom in person prior to the pandemic. During the pandemic, P32 and his family moved to a new neighborhood outside of the city where he began attending online school but with a different school district and teacher. P32 began displaying problem behavior during these transitions, particularly grabbing, and pulling others while waiting for their attention or for preferred items while attending online school and other therapies.

P49 was a 7-year-old male who displayed three forms of challenging behavior (body-hitting, grabbing others and pinching others). He used a combination of simple sign language, vocal language with 1-word requests, and picture exchange communication system (PECS) to communicate, with the PECS system being the primary communication method utilizing only one picture exchange. Prior to the pandemic, P49 attended school in person in a special education classroom and attended a clinic to receive 20 h of ABA therapy in person. During the pandemic, however, P49’s ABA therapy was paused and he was not receiving any therapy at the time of the follow-up study.

##### Measures

Caregivers were administered the *Open-Ended Functional Assessment Interview* [27] to identify the potential sources of reinforcement for challenging behavior. The interview contains questions concerning antecedents and consequences for challenging behaviors and was conducted with a Board Certified Behavior Analyst (BCBA) via phone call or Zoom videoconference call, depending on caregiver preference. The interview takes approximately 1 h.

The *Treatment Acceptability Rating Form–Revised* (TARF-R) [28] was administered to measure parent treatment satisfaction. The 21-item scale resolves in 6 subscales: willingness, reasonableness, effectiveness, cost, side effects, and disruptiveness. Scores on each subscale range from 3 to 21 with high scores on the willingness, reasonableness, and effectiveness subscales and low scores on the cost, side effects, and disruptiveness subscales indicating high treatment acceptability. The TARF-R scale has good internal consistency with Cronbach’s alpha coefficients ranging from .85 to .95 with a mean of .90.

Finally, the BPI-S was administered prior to the booster session and 4 weeks following the booster session to measure the frequency of the child’s challenging behavior.

##### Environmental modifications

All participants received the booster session in their homes with minimal environmental modifications required. P10’s mother was advised to remove breakable items from the environment and to have access to the family’s security camera system to be able to remove attention fully while ensuring child’s safety. P10 and P14 were advised to cover the tablet screen to prevent child distractions.

##### Materials

Each parent-child dyad was required to have an electronic device with a camera and audio (e.g., smart phone, tablet, or computer). Most parent-child dyads still had the Bluetooth earpiece (e.g., Axgio, Tronfy) provided in the previous RCT study and were able to utilize it to minimize distractions to the child. Each parent was provided with a video conferencing link to a HIPAA compliant Zoom room under Stanford University’s technology policy. Booster and post-booster sessions were recorded via the Zoom cloud and stored in Box, a HIPAA compliant software storage system for later coding.

The booster parent coaching session was provided to each primary caregiver by a Board Certified Behavior Analyst (BCBA) via telehealth. The booster parent coaching session was 1 h in duration and consisted of 2 components: (1) a 30-min PowerPoint presentation, and (2) a 30-min treatment implementation with live coaching with their child. During the presentation, the BCBA reviewed the steps that had previously been trained in the RCT study. After the presentation, the BCBA coached the caregiver to implement the steps with the child providing live guidance and step correction as needed for errors of omission or commission.

After 1 week of completing the booster session, a 1-h post-booster session was provided to the caregiver via telehealth. During this post-booster session, the BCBA asked the caregiver to implement the protocols covered during the booster session without reviewing the protocols and without live coaching. The BCBA collected data on parent treatment fidelity and child challenging behavior. If the parent procedural fidelity scores did not reach 80% during the post-booster session, the BCBA would review any errors during protocol implementation with the caregiver and would offer a second post-booster session. However, none of the caregivers made errors during the post-booster, and they were all able to implement treatment with 100% fidelity.

##### Treatment procedures

During FCT treatment, procedures were matched to the sources of reinforcement identified from the Open-Ended Functional Assessment Interview. For challenging behavior maintained by social-positive reinforcement (access to tangible items or attention), the caregiver was coached to sit near the child in the same room with moderately preferred toys and provide them with attention or the highly preferred tangible item for 30 s. The caregiver was then coached to provide a natural warning that the attention or the tangible item was going to be unavailable (e.g., “I have to cook dinner”, “You’ll have to put the iPad away”). If the caregiver had more time available to engage with the child, or the child was allowed to play with the tangible item, the caregiver was coached to provide a reminder to the child (e.g., “Remember, we may have some extra time to play if you’d like”). However, if there was not more time available, the caregiver was coached to provide the child a reminder on when he could access the attention or tangible again in the future (e.g., “I can play with you again after dinner”, “You can play more with the iPad after dinner”). The caregiver was coached to prompt any communication when more time was available if the child didn’t request the attention or tangible item independently and to prompt the child to engage with alternative and moderately preferred items while waiting for the attention or highly preferred tangible item, simultaneously providing a natural visual signal (e.g., one finger up, timer). If the child displayed any problem behavior, the caregiver was coached to ignore and block it, while providing the natural visual signal for the waiting skill. If the functional communication response occurred independently or without problem behavior, the caregiver was coached to provide the corresponding reinforcement (attention or tangible) for 20 s with social praise.

For challenging behavior maintained by social-negative reinforcement (escape from academic or transition demand), the caregiver was coached to make the necessary environmental modifications for safety purposes and provide a moderately preferred item to the child without presenting any demands for 30 s. Once 30 s had elapsed, the caregiver was coached to provide a natural warning to complete a task or to transition to another activity (e.g., “put away your toys so you can do your homework”, or “5 more seconds of TV and then you’ll go wash your hands”). After the child had completed the task, the caregiver was coached to provide praise for task completion and to provide a reminder that the child could have a break (e.g., “You did it! That was great work, you can have a break if you’d like”). If there was more time available for the child to engage with the current activity, the caregiver was coached to provide a natural reminder to the child of more time available (e.g., “Remember, we may have some extra time to continue the same activity if you’d like”). If the child displayed any problem behavior, the caregiver was coached to ignore and block it, while providing a natural visual signal for the task or transition completion skill. If the functional communication response occurred independently or without problem behavior, then the caregiver was coached to provide the corresponding reinforcement (break from academic or transition demand) for 20 s with social praise.

### Results

The mean age of the participants in study 2 was 10.35 years (SD = 2.0 years) and the mean expressive communication age equivalent on the Vineland Adaptive Behavior Scales, 3rd Edition [29] was 2.7 years (SD = 1.4 years). All six boys were taking psychotropic medications. The functions of challenging behavior identified from the Open-Ended Functional Assessment Interview are shown in Table 3. Table 3 also shows the forms of challenging behavior reported to occur daily prior to the booster and at the 4-week follow-up (post-booster).

**Table 3:** Characteristics of boys with FXS included in study 2

	Age (years)	Expressive communication age (years)^a^	Psychotropic medications	Other treatments	Function(s) of challenging behavior identified^b^	Forms of challenging behavior on daily basis^c^	
						Pre-booster	Post-booster
P10	11	2.2	Aripiprazole Fluoxetine	Speech therapy (.5 h/week) Behavior therapy (2 h/week)	Attention Transition escape	Hitting others Kicking others Grabbing others Destroying things	None
P14	9.11	3.8	Dextro-amphetamine	Speech therapy (1 h/week) Physical therapy (.5 h/week) Occupational therapy (.5 h/week) Behavior therapy (1 h/week)	Attention Tangible	Self-biting Head-hitting	Self-biting Head-hitting
P23	11.8	1.5	Quetiapine Clonidine Risperidone	Speech therapy (.5 h/week) Occupational therapy (.5 h/week) Behavior therapy (2 h/week)	Attention Academic escape	Self-biting Head-hitting Hitting others Grabbing	Self-biting Hitting others Grabbing others
P25	13	3.5	Fluoxetine Clonidine	None	Transition escape	Self-biting Self-scratching Hitting others Kicking others Biting others Destroying things	Self-biting Self-scratching
P32	9.8	3.1	Methylphenidate Aripiprazole Lacosamide Baclofen	Speech therapy (1 h/week) Occupational therapy (1 h/week) Physical therapy (1 h/week)	Attention Tangible	Grabbing others Verbally abusive	Grabbing others verbally abusive
P49	7.4	.11	Clonidine Guanfacine	None	Attention Academic escape	Body hitting Grabbing others Pinching others	Destroying things

^a^ Vineland Adaptive Behavior Scales, 3rd Edition

^b^ Open-Ended Functional Assessment Interview

^c^Behavior Problems Inventory, Short Form

In terms of the functions of challenging behavior identified, five of the six participants displayed two functions of challenging behavior. In all five cases, one of the functions was to gain access to attention. The median number of forms of challenging behavior displayed daily prior to the booster session was 3.5 (range = 2 to 6). At the 4-week follow-up following the booster session, the median number of forms of challenging behavior reported decreased to 2 (range = 0 to 3). Four of the 6 participants evidenced a decrease in the number of forms of challenging behavior displayed, with one participant (P10) no longer showing any forms of challenging behavior on a daily basis following the booster. For two participants (P14 and P32), the forms of challenging behavior reported daily stayed the same. These data indicated that the booster session may have facilitated implementation of the intervention for at least four of the six cases and led to subsequent improvements in their behavior.

Table 4 shows the data obtained on the TARF-R for each caregiver following the booster session.

**Table 4:** Treatment acceptability ratings

	TARF-R subscale ^a^					
	Reasonableness	Effectiveness	Side effects	Disruptiveness	Cost	Willingness
P10	19	12	3	3	3	21
P14	19	17	7	6	3	18
P23	18	18	6	9	3	21
P25	21	20	6	10	3	20
P32	17	18	4	11	3	18
P49	19	14	3	6	3	16
Mean	18.83	16.5	4.83	7.5	3	19

^a^Treatment Acceptability Rating Form-Revised

Scores on the reasonableness, effectiveness, and willingness subscales were high, indicating that the acceptability of the treatment was good. Scores on the cost and side-effects subscales were low, indicating that those factors did not impact the acceptability of the treatment. The score on the disruptiveness subscale indicated that caregivers did not find the treatment to be disruptive to the family.

### General discussion

The goal of the current study was to conduct a follow-up of families included in our previous study who had received either FCT treatment or continued with treatment as usual to examine the longer-term effects of administering FCT treatments for challenging behaviors in young boys with FXS. Results showed that boys who had previously received 12 weeks of FCT via telehealth were more likely to show improvements in rates of challenging behavior over the follow-up interval than the boys who had continued with treatment as usual. Admittedly, the mean change in score on the irritability subscale of the ABC-C was relatively small (a decrease of 2 points per year). However, given that the mean follow-up time was 3 years, this would equate to a decrease in score of 6 points over that period. Given that the mean score on the irritability scale on the ABC-C at baseline in our RCT was 19.5 points, this amounted to a 30% decrease. These data suggest that implementing behavioral treatments that empower parents with the strategies to manage their child’s challenging behavior can result in the maintenance of treatment effects over several years.

Differences were also found between the groups in terms of changes in levels of parental stress. Both groups of caregivers experienced high levels of parental stress at baseline in the previous study, but these stress levels decreased at a faster rate over the follow-up interval for those who had received FCT treatment. One potential factor that could have influenced these results is the onset of the COVID-19 pandemic. Our previous study was conducted between 2016 and 2019 (i.e., pre-pandemic) whereas the present study was conducted approximately 1 year into the pandemic (March–August 2021). The number of hours spent at home, socially distanced from others, would therefore have dramatically increased in all families at the follow-up point. However, families who had participated at the beginning of the previous study in 2016 (and therefore had longer follow-up times) would have experienced most of the follow-up period under “normal” pre-pandemic conditions whereas families who had participated at the end of previous study in 2019 (and therefore had shorter follow-up times) would have experienced the majority of their follow-up period under pandemic conditions (i.e., working and caring for their children at home, often without childcare due to social distancing). The results of the study therefore need to be set in this context. Interestingly, we found that boys who were followed-up after a shorter time lapse exhibited greater decreases in challenging behavior over the follow-up interval. These data provide additional support for implementing booster sessions on a regular basis.

At follow-up, we found that 11 of 16 boys with FXS who had received FCT treatment and 14 of 18 boys who had received treatment as usual still exhibited at least one form of challenging behavior on a daily basis. For participants in the FCT treatment group, we invited families to receive a subsequent booster training session. We also interviewed caregivers to identify the potential function(s) of their child’s challenging behavior. Six of the 11 families agreed to participate in the booster training and four of the six boys evidenced a decrease in the number of forms of challenging behavior displayed daily following the booster training. Although these effects were important, the benefits of the booster training were likely limited due to the short duration of the training as well as the short follow-up time (4 weeks). Future research is warranted to determine optimal titration of treatment dosage for long-term maintenance. For example, older patients with a prolonged history of reinforcement may benefit from monthly parent booster sessions for 6 months, prior to discharge. The aim of this type of titration model would be to prevent challenging behaviors from increasing back up to daily occurrences. Future studies will need to determine the optimal length of the booster training and whether further booster sessions are needed to optimize the treatment effect.

The results of the functional assessment interview conducted prior to the booster session indicated that in 5 of 6 cases, the child’s challenging behavior was maintained by multiple sources of social reinforcement. In all 5 of those cases, one of the sources of reinforcement was to obtain attention from caregivers. A limitation of the current study was that we were unable to conduct a formal in-home functional analysis of the child’s challenging behavior. This was primarily due to restrictions imposed by the pandemic. However, in all cases, the functions identified in the current study were similar to the functions of challenging behavior identified in our previous study [30]. These data indicate that the functions of challenging behavior were relatively stable across time. Future studies will need to evaluate the feasibility of conducting functional analyses via telehealth in this population.

It is also possible that the reported improvements following FCT (and possibly the improvements at follow-up) could be due to demand characteristics or other forms of bias. Specifically, the control group had previously received treatment as usual, and therefore had a very different experience in many ways from the experimental group. Caregivers who were coached to implement FCT with their child may also have had a conscious or unconscious bias to please the experimenters, resulting in greater improvements than the control group. Placebo effects in the experimental group are also possible. In future studies, it would be important to equate the demand characteristics of the two groups, for example, by asking caregivers in the control group to complete online learning modules concerning their child’s challenging behavior. These psychoeducation sessions could be conducted at a similar frequency to the treatment group. To limit potential bias in the behavioral ratings, direct observations of challenging behaviors could also be conducted using trained observers who are blinded to treatment status.

## Conclusions

This study fills a significant gap in the literature by examining the extent to which behavioral treatment effects may be maintained in FXS and further informs the field concerning the longer-term benefits and impact of behavioral treatments administered via telehealth in general. We believe that the results of our study provides valuable information on how well and how long treatment effects can be maintained and how effectively treatment effects can be restored, which has been rarely studied in the field of behavioral intervention.

## Abbreviations

FXS: Fragile X syndrome
ASD: Autism spectrum disorder
FA: Functional analysis
FCT: Functional communication training
RCT: Randomized controlled trial
BPI-S: Behavior Problems Inventory–Short Form
ABC-C: Aberrant Behavior Checklist-Community
BCBA: Board Certified Behavior Analyst
PSI-4: Parenting Stress Index, 4th Edition
TARF-R: Treatment Acceptability Rating Form-Revised
